# Addressing Public Health Emergencies via Facebook Surveys: Advantages, Challenges, and Practical Considerations

**DOI:** 10.2196/20653

**Published:** 2020-12-14

**Authors:** André Grow, Daniela Perrotta, Emanuele Del Fava, Jorge Cimentada, Francesco Rampazzo, Sofia Gil-Clavel, Emilio Zagheni

**Affiliations:** 1 Laboratory of Digital and Computational Demography Max Planck Institute for Demographic Research Rostock Germany; 2 Saïd Business School and Leverhulme Centre for Demographic Science University of Oxford Oxford United Kingdom

**Keywords:** Facebook, web-based surveys, public health emergency, COVID-19

## Abstract

Surveys of the general population can provide crucial information for designing effective nonpharmaceutical interventions to tackle public health emergencies, such as the COVID-19 pandemic. Yet, conducting such surveys can be difficult, especially when timely data collection is required. In this viewpoint paper, we discuss our experiences with using targeted Facebook advertising campaigns to address these difficulties in relation to the COVID-19 pandemic. We describe central advantages, challenges, and practical considerations. This includes a discussion of potential sources of bias and how they can be addressed.

## Introduction

As of September 9, 2020, the COVID-19 pandemic has caused over 27.4 million cases and over 894,000 deaths around the world [1]. To control the spread of COVID-19, national and local governments have implemented nonpharmaceutical interventions, including school closures, bans on large gatherings, mobility restrictions, and physical isolation, as well as unprecedented measures like local and nationwide lockdowns. Such measures have likely been critical in delaying and containing the COVID-19 pandemic [2]. However, individual behaviors, rather than governmental actions, may be crucial for controlling the spread of COVID-19 in the long run [3]. Timely and accurate data on human behaviors are thus of paramount importance in closely monitoring the adoption of preventive measures, emergence of symptoms, and changes in mobility and person-to-person contacts in the population.

In this context, surveys of the general population can provide central information needed to assess people’s acceptance of and compliance with behavioral guidelines. Such surveys are also needed to capture spontaneous bottom-up behavioral changes. Yet, researchers who want to conduct surveys that directly address ongoing epidemics are faced with unique methodological challenges, as follows: (1) these surveys need to be designed, implemented, and conducted quickly, as epidemics spread rapidly and are difficult to predict, especially when they involve new emerging diseases (ie, timeliness); (2) they need to cover the entire population, and in the event of large-scale epidemics or pandemics, they need to be conducted simultaneously in multiple countries or regions, as regional differences could be relevant for designing effective interventions (ie, coverage); and (3) they should be cost-effective, as obtaining large research funds quickly for an ad hoc survey can be difficult (ie, cost-effectiveness). Furthermore, in the case of COVID-19, the nature of recommended social distancing measures may limit some traditional modes of data collection, such as face-to-face interviews and even phone interviews, to the extent that they rely on large call centers.

In this viewpoint, we discuss the use of Facebook as a recruitment tool to address these challenges. Our assessment is based on the COVID-19 Health Behavior Survey (CHBS) that we conducted between March 13 and August 12, 2020 in 8 countries (ie, Belgium, France, Germany, Italy, the Netherlands, Spain, the United Kingdom, and the United States). Participant recruitment took place on a daily basis via targeted advertisements on Facebook, resulting in a total of 144,034 completed questionnaires. In what follows, we first provide an overview of the most important design aspects of the survey, and then discuss some of the central advantages, challenges, and practical considerations related to using Facebook advertisements in surveys that address public health emergencies, such as the COVID-19 pandemic. This includes a discussion and empirical assessment of potential sources of bias and how they can be addressed. Additionally, we make recommendations for those who want to implement similar surveys in the near future, and we hope that this will facilitate timely data collection to address the current—and possibly future—public health and societal crises.

## Methodological Approach

The CHBS is a web-based survey that focuses on people's reactions to the COVID-19 pandemic and targets individuals aged ≥18 years. The questionnaire has 4 sections, which encompass sociodemographic characteristics (eg, age, sex, and education), health indicators (eg, underlying medical conditions), behaviors and attitudes related to COVID-19 (eg, perceived threat level and preventive measures taken), and social contacts (ie, the number of interactions with other people).

Recruitment took place via Facebook, by means of advertisement campaigns that we created with the Facebook Ads Manager (FAM). The Facebook business model centers on targeted advertisements, and the FAM enables advertisers to create campaigns that can be directed at specific user groups. Targeting can be based on both users’ demographic characteristics and a set of characteristics that Facebook infers from users’ behavior on the social network. Advertising campaigns consist of 3 levels. The highest level is the campaign level, at which the goals of the campaign are defined (eg, generating awareness or generating traffic). The second level is the ad set level, at which the target audience, budget, and schedule are defined. The third level includes the advertisements themselves, which can consist of multiple advertising materials (eg, images and videos), advertising text, and the link to the page where Facebook users should be directed when they click on the ad. More details on these levels can be found in the Pötzschke and Braun [4] study.

**Figure 1:**
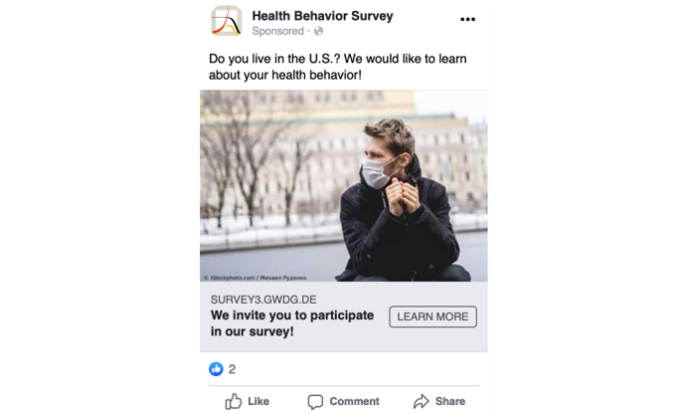
Example of an advertisement in the Facebook advertising campaign in the United States.

We created 1 campaign per country and stratified each campaign at the ad set level by users’ sex (ie, male and female), age group (ie, 18-24, 25-44, 45-64, and ≥65 years), and region of residence. In the European countries, the region classification largely followed the NUTS (nomenclature of territorial units for statistics)-1 classification, which we aggregated into larger macro regions. In the United States, the region classification was based on census regions. More details on region stratification can be found in the Perrotta et al [5] study. Our study design resulted in 24-56 strata per country. Each ad set contained 6 different images, leading to a total of 1776 different ads. Figure 1 provides an example of the ads shown to Facebook users in the United States. We launched the campaigns between March 13, 2020 (ie, in Italy, the United Kingdom, and the United States) and April 4, 2020 (ie, in Belgium). This difference in the timing of the inclusion of countries is owed to a trade-off between the time needed to translate and technically implement country-specific surveys and the goal to start data collection in a timely manner. We concluded the survey on August 12, 2020.

Facebook advertising campaigns can have different goals, and the overall costs that are incurred will partly depend on the chosen goal. We chose the goal of generating traffic. This meant that the Facebook algorithms would optimize ad delivery to maximize the likelihood of people clicking on the ad when it is shown to them. Advertisers can choose and define how Facebook should use their budget to meet these goals. For example, advertisers can set a budget that is evenly spread over a fixed period, or they can define an average daily budget that Facebook would seek to meet weekly over an unspecified period. We opted for the latter, as the duration of the COVID-19 pandemic was uncertain. Based on these parameters, ad delivery was determined through an automated bidding process, in which a given ad competes for delivery with ads from other advertisers who are targeting the same user groups. In this process, Facebook considers the budget that can be afforded for delivering a given ad and the likelihood of the ad being of interest to users by comparing it to competing advertisements. Before an ad campaign is launched, the FAM provides an estimate of various parameters, such as the size of the target audience and daily reach, which makes it possible to gauge the likely performance of the advertising campaign.

It is important to note that our study is not the first to use targeted Facebook advertisements for participant recruitment in health research. Earlier research has used this approach to address topics such as smoking behavior [6], cannabis use [7], and mental health [8]. Furthermore, Whitaker et al [9] and Thornton et al [10] have performed systematic reviews of related literature. However, compared to these studies, our survey stands out because of its cross-national character, duration, and population coverage, given that we continuously collected data from 8 countries for 5 months and recruited more than 140,000 participants from most—if not all—parts of society and subnational regions.

## Advantages

Our use of Facebook for participant recruitment enabled us to address the challenges of timeliness, coverage, and cost-effectiveness.

With regard to the advantages of our recruitment methodology, first, we were able to design, implement, and launch the survey in a timely manner. To summarize, preparing a Facebook advertising campaign involves creating an advertising account, a Facebook page that is associated with the advertisements/survey, and the ads themselves. Ad creation can be performed in bulk, by uploading a CSV (comma-separated values) file. Once the ads have been created, they need to be submitted for review, during which their compliance with Facebook advertising policies is assessed. This review can take between a couple of hours and a day, or longer. In our case, it was usually completed within 24 hours. However, it should be noted that in April/May 2020, Facebook warned advertisers that reviews could be delayed due to the COVID-19 pandemic [11]. Once reviewed, the ads can be delivered. Ads only need to be reviewed again when major changes are made (eg, changes to the advertising materials or ad text).

The second advantage is that our use of Facebook enabled us to draw multinational samples from diverse parts of the respective national populations. Facebook is the largest social media platform, with 2.45 billion monthly active users worldwide as of Fall 2019 [12]. In the United States, about 68% of adults used Facebook in 2018, whereas this percentage is 56% in Germany, 75% in Italy and Spain, 76% in France, 79% in the Netherlands, 85% in the United Kingdom, 89% in Belgium [13,14]. Compared to younger and middle-aged adults, older adults generally tend to be underrepresented on Facebook [15]. Nevertheless, the Facebook user population provides a cross section of the overall population with access to the internet. A comparison of different social media platforms (ie, Facebook, LinkedIn, Twitter, Tumblr, and Reddit) in the United States has suggested that Facebook is the most representative in terms of users’ educational attainment and internet skills [16].

The third advantage is that our use of Facebook made participant recruitment comparatively cost-effective, even though it can be difficult to determine the exact costs in advance. This is partly due to the nature of the bidding process that determines ad delivery and variation in the competition for advertising space. A central performance measure of Facebook advertising campaigns is the cost per click (CPC) value. Whitaker et al [9] performed a review of studies that used Facebook for recruiting participants in health research, and they reported CPC values between €0.17 (US $0.20) and €1.46 (US $1.74). This variation is likely due to differences in the definitions of targeted user groups, the competition from other advertisers, and the likelihood of users clicking on the respective ad. Our costs were similar to those reported in earlier research. Between March 13 and August 12, 2020, we collected 144,034 questionnaires at an overall CPC of about €0.14 (US $0.17) and an overall cost per completed questionnaire (CPCQ) of about €1.05 (US $1.25), excluding the value-added tax. It should be noted that as a relief measure, the value-added tax in Germany was temporarily reduced from 19% to 16% on July 1, 2020. The difference between the CPC and CPCQ is due to Facebook users who clicked on one of our ads, but did not complete our questionnaire.

**Figure 2:**
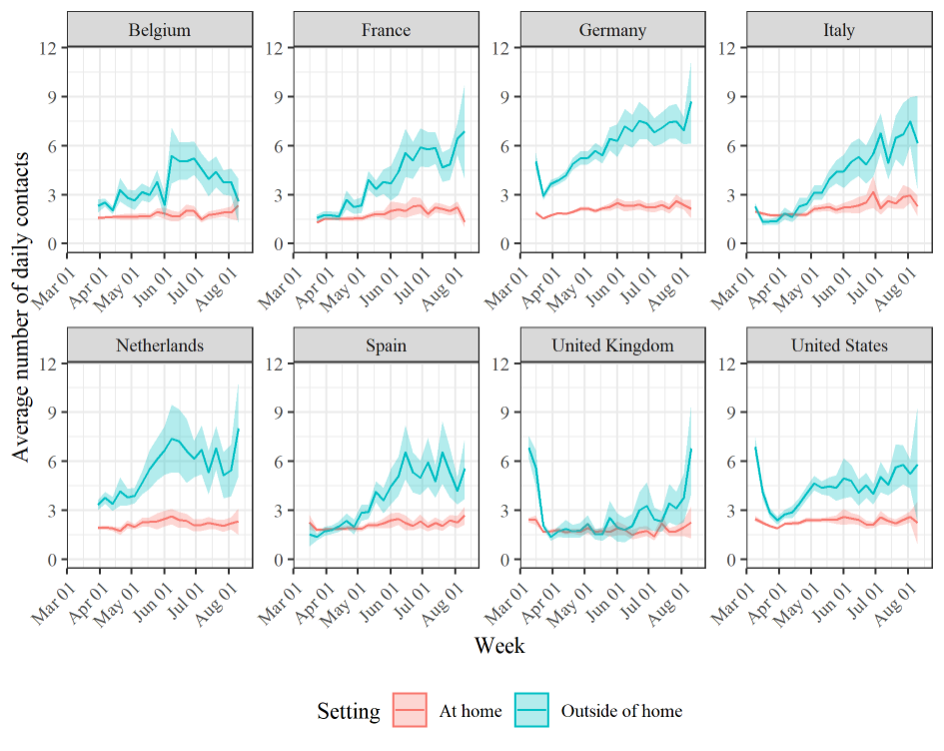
Average number of contacts at home and outside of home per week between March 13 and August 12, 2020 across the 8 countries. Lines show averages and shaded areas show 95% confidence intervals. Poststratification weighting has been applied.

Thanks to these advantages, we were able to collect data that provided key insights into attitudes and behaviors that shape—and are shaped by—the COVID-19 pandemic. Figure 2 illustrates this by plotting the average number of face-to-face social contacts that respondents reported for the day before participating in the survey for the entire observation period. Face-to-face social contacts are the main vehicle for virus spread, given that the SARS-CoV-2 virus is mainly transmitted by infected secretions or respiratory droplets [17]. Figure 2 shows that there was great variation in the number of face-to-face contacts over time, especially in those that occurred outside the home. When paired with external information on the COVID-19 pandemic, such as lockdown measures and infection rates, these data provide valuable insights into the effectiveness of different policies. When the data is further broken down by respondents’ demographic characteristics, it also becomes possible to assess whether different demographic groups respond differently to different policies. The data can also be used to calculate central epidemiological metrics (eg, the effective reproduction number, Rt) and design more realistic epidemiological models. More details on the contact patterns that we observed in the CHBS can be found in the Del Fava et al [18] study. Insights into other behaviors and attitudes toward the COVID-19 pandemic can be found in the Perrotta et al [5] study.

To better illustrate the comparative strengths of our approach, it is helpful to contrast our work with similar ad hoc web-based survey efforts that also focus on COVID-19. A prominent example is the work of Fetzer et al [19], who used an open survey to recruit 108,075 participants from 58 countries between March 20 and April 7, 2020. In this study, recruitment took place via link sharing on social media and similar channels. Another prominent example is the work of De Coninck et al [20], who used an existing opt-in online panel that was maintained by a commercial polling company to recruit 1000 participants in the Flemish region of Belgium between March 17 and March 22, 2020. Both studies used web-based surveys to collect information about people’s attitudes and behaviors toward COVID-19. Compared to Fetzer et al [19], our use of Facebook offered more control over the recruitment process, given that when a survey link is shared via social media, it is not possible to control who is invited to participate in the survey. Hence, our targeted advertising methodology made it arguably easier to ensure that our samples were demographically balanced. Compared to De Coninck et al [20], our use of Facebook arguably offered less control over the recruitment process, given that existing online panels typically offer more detailed information about prospective participants than the FAM. These panels make it easier to collect demographically balanced samples. However, our use of Facebook offered a larger reach in terms of the number of countries that could be included and the time frame that was covered. In terms of costs, the CPCQ that our paid advertisements incurred was higher than the CPCQ incurred by Fetzer et al [19], as their approach to link sharing did not incur any costs. However, based on our personal communication with De Coninck et al [20], the CPCQ we incurred was similar to that of De Coninck et al [20], who paid a commercial polling company for data collection.

## Challenges

Eliciting information via self-administered web-based surveys involves several challenges. For example, issues with recall inaccuracy often occur when some time has elapsed between a specific event of interest and participation in the survey [21,22]. This puts limits on the information that can be collected, and such issues should therefore be considered in light of the goals of the respective study. If, for example, detailed and accurate medical information is essential (eg, exact blood pressure measurements), a web-based survey may not be the best choice, and an in-person assessment with medically trained personnel may be preferable. Discussing the methodological challenges of web-based surveys is out of the scope of this paper. Instead, we focus on the challenges that are specific to using Facebook for participant recruitment. A discussion on the methodological challenges of web-based surveys can be found in the Eysenbach and Wyatt [23] study.

The most important set of challenges relates to the issue of self-selection bias. The Facebook user base is a rough cross section of the overall population with internet access, but not all demographic groups are equally well represented [24]. Additionally, there may be variation in Facebook users’ interest in the survey topic. Hence, there is no guarantee that the resulting samples will be representative in terms of central demographic characteristics (eg, age and sex) and important unobservable characteristics. This issue is potentially exacerbated by the algorithmic optimization that Facebook uses for ad delivery. If certain demographic groups are more likely to click on an ad than others, Facebook might increasingly deliver the ads to these groups, thereby reinforcing existing self-selection bias. This is particularly difficult to correct if survey participation and ad delivery are affected by user characteristics that cannot be easily considered when defining the relevant sampling strata.

It is important to note that if a survey is conducted over a long period of time, there may be changes in Facebook user activity. This may be due to seasonal variation in people’s use of Facebook [25]. However, it seems possible that the development of the COVID-19 pandemic may have also led to changes in the composition of our samples over time. In the early days of the pandemic especially, the SARS-CoV-2 virus dominated the news, and lockdown measures were put in place to curb its spread. This may have increased participation in our survey in 2 ways. First, a lack of alternative activities due to lockdown measures may have led people from various sub-populations to spend more time on Facebook than normal, and this may have increased the likelihood of seeing our ads. Second, the salience of the pandemic may have increased the chance of people clicking on our ads when seeing them. Over time, as the number of infections decreased and lockdown measures eased, participation in the survey may have decreased, and the resulting samples may have become more selective.

**Figure 3:**
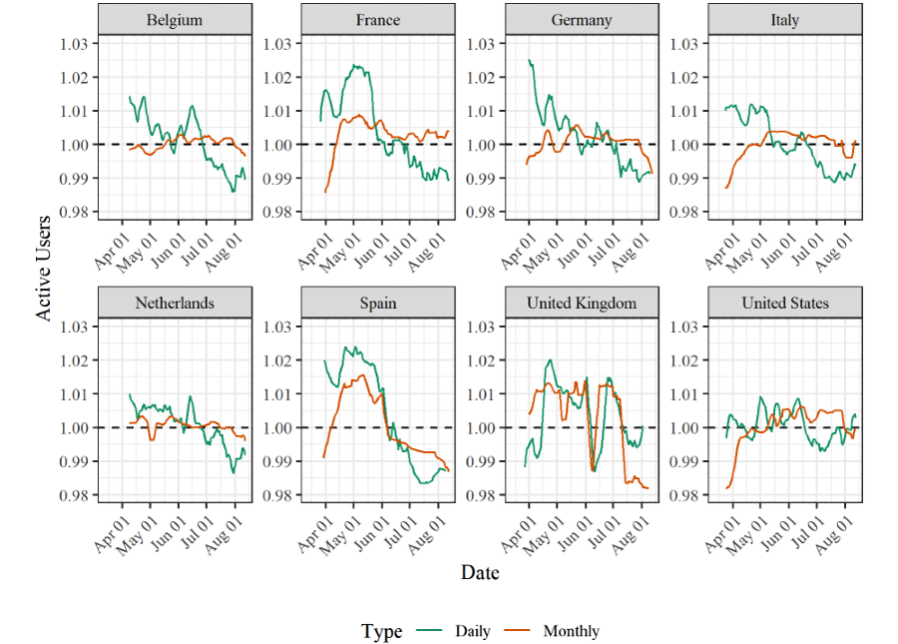
Number of DAUs and MAUs between March 13 and August 12, 2020 across the 8 countries based on all ad sets. Numbers were standardized for each country by dividing the value for a given day by the average number of DAUs/MAUs over the entire observation period. Lines show 7-day moving averages. We collected DAU and MAU values every 6 hours, as these estimates can change within 1 day. We averaged these multiple observations to obtain 1 number per day. Data collection started between March 18 and April 4; no data was collected on April 11, April 12, and between August 3 and 6 due to technical issues. DAU: daily active Facebook user; MAU: monthly active Facebook user.

**Figure 4:**
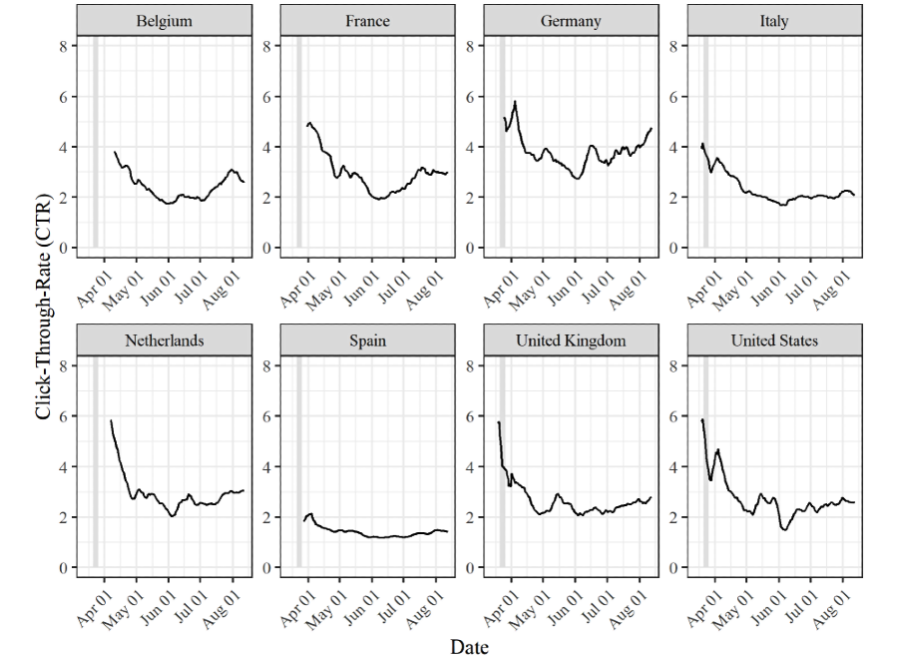
CTRs between March 13 and August 12, 2020 across the 8 countries. The line shows the 7-day moving average. The shaded area shows the period during which technical problems with ad delivery occurred. CTR: click-through rate.

Figures 3 and 4 show changes in user behavior and survey participation over time. This was done by plotting the average number of daily active Facebook users (DAUs) and monthly active Facebook users (MAUs), as well as the click-through rates (CTRs), for all countries over the entire study period. All 3 measures are based on estimates from Facebook, which we obtained via the Facebook application programming interface. The number of DAUs is the number of unique active users on a given day, whereas the number of MAUs provides the number of unique users who have been active on Facebook within the last 30 days [26]. Both estimates are commonly used to assess the potential reach of advertising campaigns, and we systematically collected DAU and MAU estimates for all our strata over the entire study period. The CTR is defined as the number of people who click on an ad after seeing it, and CTRs become available after a campaign has been started and delivered to users [27]. Hence, changes in the number of DAUs and MAUs provide insights into changes in Facebook use, whereas changes in the CTR provide insights into topic salience/interest among Facebook users. It should be noted that between March 21 and March 26, 2020, we experienced technical problems with ad delivery across several countries, leading to a substantially lower number of participants than in the other weeks of our study. The CRT values for this period are therefore less reliable than those for the rest of the survey period.

As shown in Figure 3, the number of DAUs was largest during the early weeks of the observation period for most countries. However, this number gradually decreased, usually by 3-5 percentage points. This means that the number of unique users who could have seen our ads on a given day decreased over time. The only exceptions to this were the United Kingdom and the United States, where changes in the number of DAUs were more erratic. The trends in the MAU values somewhat deviate from those in DAU values. Typically, there was an initial increase in the number of MAUs, but this number later decreased. Hence, while the number of individuals who may have seen our ads on a daily basis decreased over time, we may have reached users who we would not have reached in other months toward the middle of the observation period. Furthermore, in Figure 4, the CTR values show a clear trend over time. The CTR was initially high across all countries, and then it decreased before ultimately increasing again. This means that in the early phases of the survey, Facebook users were more likely to click on our ads than in later phases. Hence, our data suggest that over time, the process by which users selected themselves into the survey may have changed, but the data do not allow us to precisely assess why these changes occurred.

How can the problem of self-selection be addressed? We suggest 4 methodological steps that can help alleviate this problem. First, in line with common approaches in traditional survey research, we suggest stratifying ad campaigns based on characteristics that are known to relate to survey participation and the outcome of interest, such as in the Pötzschke and Braun [4] study. Evidently, in the case of new emerging diseases, relevant individual characteristics are difficult to know in advance. Moreover, several relevant characteristics will not be available for creating strata in the FAM (eg, preexisting medical conditions). However, characteristics that are available (eg, age and sex) should be considered for stratifying advertising campaigns. We also suggest stratifying ads by region within countries, as people’s responses might vary locally. With this approach, the bias that Facebook’s ad delivery algorithms may generate is counteracted, leading to more balanced samples.

The second step we suggest, which is in line with the Zagheni and Weber [28] study, is applying poststratification techniques to the samples obtained from Facebook, to the extent that they deviate from the overall population in terms of important characteristics. In this regard, using Facebook offers distinct advantages over other, less controlled ways of recruiting online samples. As indicated previously, prior to launching a campaign, the FAM provides an estimate of the size of the audience with the characteristics of interest. Arguably, this feature is similar to a sampling plan and has been used in earlier research to conduct a “virtual census” of the overall population, such as in the Zagheni et al [29] study. Furthermore, the ad performance estimates that the FAM provides after a campaign has been launched (eg, the number of users to whom a given campaign, ad set, or ad has been delivered and the number of users who have clicked on the ad) can be paired with information about survey completion rates for each stratum. This makes it possible to calculate performance measures, such as approximate participation rates. However, it is important to keep in mind that many of the measures that Facebook reports are only estimates. The resulting indicators should thus be viewed as informed proxies.

As a complement to this approach, Zhang et al [30] recently reported that by selectively activating and deactivating ad sets over the course of the survey period, it is possible to obtain representative samples from Facebook that do not require poststratification. This approach is feasible if the specific timing of participation over the study period does not matter. However, this was not feasible in our case, as our goal was to obtain daily balanced samples. Selectively closing and opening ads over the course of a day or week would have implied that responses from certain subgroups may have been concentrated during a certain time of the day or certain days of the week. As an alternative, researchers may opt for dynamically adjusting the budget, so that more money is spent on strata that are underrepresented in the survey. With this approach, it is important to keep in mind that large changes in the budget allocated to an ad set may trigger the ad review process again, which can lead to a gap in data collection. As this would have undermined the goals of our study, we decided against this approach. Instead, we continuously recruited members of all strata using a stable budget.

With regard to the third step, we suggest that the issue of self-selection due to participant characteristics that are difficult to observe before people take part in the survey can be partly addressed by considering possible sources of bias in the design of the survey and advertising campaign. In the case of our study, we expected that individuals who are particularly concerned about COVID-19 might be more likely to participate, and such concerns might also be reflected in reported behaviors and attitudes. As it is not possible to stratify Facebook advertising campaigns based on such concerns, we considered this issue in 2 ways. First, in the survey, we directly assessed participants’ concerns about COVID-19 and other factors that may raise such concerns. Second, when selecting the images for our ads, we aimed to create variation in how closely the images were linked to the topic of COVID-19. In our analyses, we were able to control the extent of how all these factors affected participation and answers to other questions.

**Figure 5:**
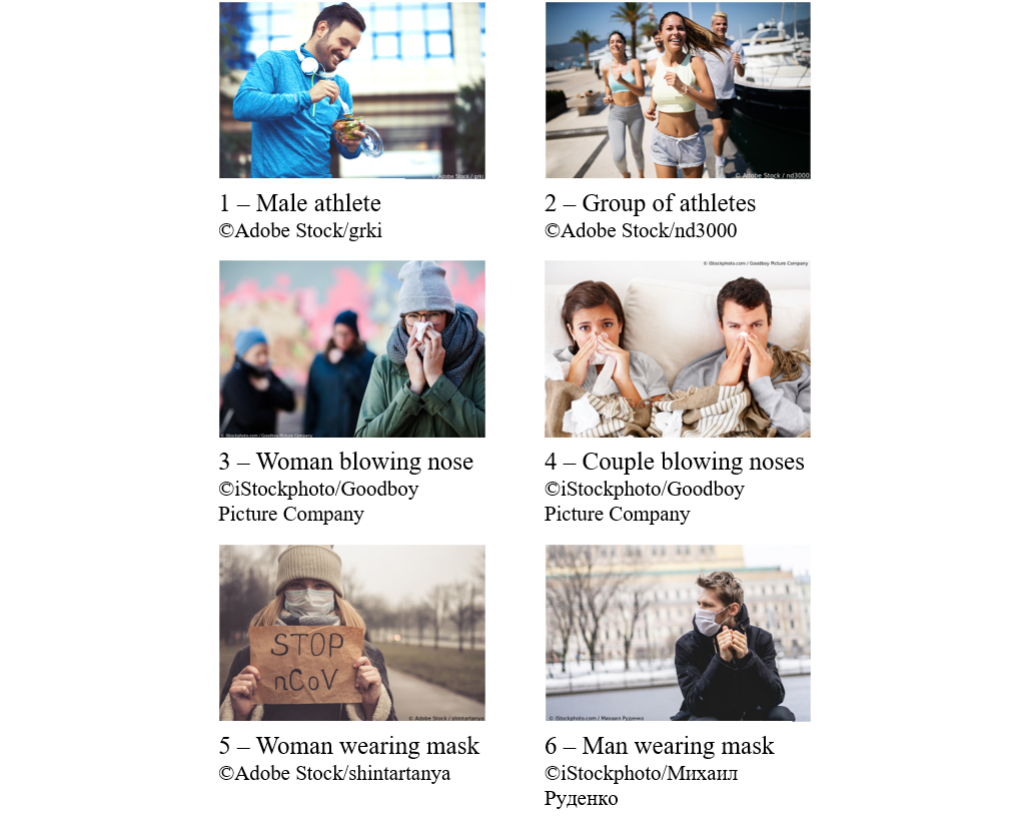
Images used in the Facebook advertising campaigns.

Figure 5 shows the different images that we used in the ads. We considered images 1 and 2 to be the least strongly linked to COVID-19 and images 5 and 6 to be the most strongly linked to COVID-19. In total, 141,879 of the respondents arrived at the survey via one of the ads. Of these respondents, about 74% (104,292/141,879) arrived via image 5, about 16% arrived via image 6 (23,157/141,879), about 7% (9699/141,879) arrived via image 3, and the rest arrived via the remaining images. To assess whether the image through which participants arrived at the survey was related to their concerns about COVID-19, we conducted a Kruskal-Wallis test by rank. In this test, we assessed respondents’ personal perceptions of how large a threat COVID-19 presented for themselves. This was determined using a 5-point Likert-type scale, in which a score of 1 represents very low threat and a score of 5 represents very high threat. These scores were associated with the picture through which participants arrived at the survey. Table 1 shows the number of respondents who selected 4 or 5 on the scale, which indicated a high or very high threat perception, respectively. The number of respondents who perceived COVID-19 as a high or very high threat was largest among those who arrived at the survey via image 3 (7178/9280, 77%) and lowest among those who arrived via image 2 (922/1461, 63%). The observed variation in threat perceptions across images was significant at the 1% level (*χ²*_5_=801, *P*<.01). A broader analysis of how the different images related to respondents’ self-reported attitudes and behaviors would be important, but our assessment suggests that the inclusion of different images helped with recruiting more diverse samples in terms of concerns for COVID-19.

With regard to the fourth step, in recent years, the multilevel regression and poststratification approach to making inferences from highly selected survey data [31] has proved effective in producing unbiased population estimates [32,33]. In the first stage of multilevel regression and poststratification, the sample is partitioned into a large number of demographic strata (eg, each combination of age group, sex, and region), and a multilevel regression model is used to estimate the outcome of interest, such as the average number of contacts or the percentage of people wearing a face mask, in each stratum. In the second stage of multilevel regression, the stratum-level estimates are used to produce a final population-level estimate, and poststratification weights are used to account for the proportion of each stratum in the population. This approach combined with the previously mentioned steps, and a fine partition of the sample in demographic strata enables researchers to make proper inferences at the population level, even in presence of strong selection bias.

**Table 1:** The number of respondents who personally perceived COVID-19 as a high (score=4) or very high threat (score=5) to themselves based on a 5-point Likert-type scale for each ad image. No weighting has been applied.

Image number	Image name	Total, n/N (%)
1	Male athlete	422/586 (72%)
2	Group of athletes	922/1461 (63%)
3	Woman blowing nose	7178/9280 (77%)
4	Couple blowing noses	1819/2519 (72%)
5	Woman wearing mask	69,421/102,061 (68%)
6	Man wearing mask	16,882/22,451 (75%)

Another challenge relates to trust in web-based surveys. Web-based surveys may face suspicion, as they could be used to elicit personal information for nonresearch purposes (eg, marketing, identity theft, etc) [34]. In addition, over the last several years, there have been several incidents that may have negatively affected the trust that the public has in the data protection measures put in place by Facebook. A prominent example is the Cambridge Analytica scandal, in which the personal data of Facebook users was harvested without consent, with the goal of influencing the 2016 US presidential election through microtargeting [35]. When fielding our survey, we encountered such suspicions in the commenting sections of our ads, and 1 notable concern was that Facebook would transfer personal user information to us. We addressed this issue by highlighting that the survey was anonymous and that no personal information was exchanged with Facebook. We also provided additional information about our research institute, the research team, and the goals of our survey by providing a link to our data protection policy and providing information about preliminary results and reports as they became available. Despite these measures, it seems likely that Facebook users who are concerned about data privacy were less inclined to participate in our survey. Additionally, while anonymous web-based surveys have the potential to reduce the likelihood of respondents providing socially desirable answers compared to personal interviews [36], privacy concerns may have rendered them reluctant to answer questions that they perceive as sensitive. We addressed this issue by offering the possibility of not answering questions that they feel uncomfortable with, to avoid forcing answers on sensitive topics.

## Practical Considerations

There are some practical aspects that need to be considered when using Facebook ads for survey research. First, the possibility of targeting certain user groups makes it easier to recruit members from certain subpopulations, even when they are underrepresented on Facebook. Yet, it is important to keep in mind that as the number of strata in the campaign increases, so will the selectivity and costs of the campaign. This is particularly true if the members of certain strata are less likely to participate than members of other strata. For example, if the goal is to stratify a campaign based on 5-year age groups, the number of strata will increase considerably compared to strata based on 10- or 20-year age groups. This means that overall, more responses will need to be collected to have enough observations per stratum to apply poststratification weighting. Furthermore, when the members of certain groups engage little with Facebook, a larger share of the budget needs to be devoted to recruiting them.

Second, both the advertisements and the study page to which these advertisements are linked need to be actively managed. Facebook advertisements are similar to user posts, meaning that users can react to them (eg, liking), comment on them, and share them with friends. Additionally, users can leave posts on the study page and review the page itself. In our experience, it is important to engage with user comments and provide additional information if needed, to maintain trust with current and prospective participants. The time investment that this requires should not be underestimated, especially for studies that run for a long period of time and are conducted in multiple languages. On average, our ads received about 19,300 impressions and about 135 comments per day, and it took us about 1 hour to manage 50-100 comments. The exact time it took to manage comments depended on the length of the comment and the complexity of the answer that was required. Over time, we became more experienced and efficient in managing comments. Based on these numbers, researchers who want to conduct a study on a similar topic and receive a similar number of impressions per day should expect to spend about 1-2.5 hours per day managing comments.

Third, the ad review process involves an automatic assessment of the links that are provided in the ads. In this process, the webpage to which the link leads is accessed. Hence, submitting ads for review can generate a large amount of traffic for a web-based survey, and it is important to keep in mind that the number of times that the survey page was accessed is not equivalent to the number of potential respondents who have accessed it, as some of the traffic may have been generated by the review process. It would therefore not be valid to approximate the survey completion rate by dividing the number of completed questionnaires by the number of page accesses. Furthermore, it is important to schedule sufficient time between submitting ads for review and launching the data collection. In our case, the review process was usually completed in a timely manner. However, there might be delays (eg, those predicted by Facebook due to COVID-19) and problems (eg, rejections due to violations of the Facebook advertising policies) that increase the time between review completion and delivery.

## Conclusion

To conclude, we suggest that targeted advertisements on Facebook can be a powerful tool for recruiting participants in ad hoc surveys of the general population during a public health emergency, as long as certain methodological steps are taken to address the issue of self-selection. We hope that the experiences that we have described here, together with our recommendations, will make it easier for other researchers to implement similar surveys that tackle current and future pandemics.

## Abbreviations

CHBS: COVID-19 Health Behavior Survey
CPC: cost per click
CPCQ: cost per completed questionnaire
CSV: comma-separated values
CTR: click-through rate
DAU: daily active Facebook user
FAM: Facebook Ads Manager
MAU: monthly active Facebook user
NUTS: nomenclature of territorial units for statistics
